# The complete chloroplast genome sequence of *Manglietia yuyuanensis* (Magnoliaceae)

**DOI:** 10.1080/23802359.2019.1703568

**Published:** 2020-01-08

**Authors:** Junqing Wang, Peng Li, Dongdong Mao, Xiaomin Zhang, Huan Wang, Yanyan Li

**Affiliations:** Chemical and Environmental Engineering College of Pingdingshan University, Pingdingshan University, Pingdingshan, Henan, P. R. China

**Keywords:** *Manglietia yuyuanensis*, chloroplast genome, Magnoliaceae, phylogenetic analysis

## Abstract

*Manglietia yuyuanensis* is an important afforestation and excellent broad-leaved tree species in southern China. In this study, we assembled the complete chloroplast genome of *M. yuyuanensis* based on the Illumina sequences, sequence analysis showed the genome was 160,078 bp in length presenting a typical quadripartite structure and contains an inverted repeat region (IR, 26,467 b), a small single-copy (SSC) region, and a large single-copy (LSC) region (18,785 and 88,359 bp, respectively). The overall GC content was 39.27%. The sequence contained 128 unique genes, including 81 protein-coding genes, 38 tRNA genes, and 8 rRNA genes. The maximum-likelihood (ML) phylogenetic analysis revealed that *M. maudiae* was closely related to *Manglietia insignis*. A phylogenetic analysis revealed that *M. yuyuanensis* is closely related to *Manglietia glaucifolia*, with the genus *Manglietia*.

*Manglietia yuyuanensis* is an important afforestation and excellent broad-leaved tree species with high scientific and economic values in southern China, belonging to the Magnoliaceae family, and therefore, has always been cultivated for wood production. The Magnoliaceae is considered as one of the most primitive groups of angiosperms (Li and Guo 2014). The family includes two separate lineages. The first, *Liriodendroideae*, comprises the genus *Liriodendron* with only two species, whereas the second, *Magnolioideae*, is thought to include one genus *Magnolia* (Nooteboom 2000; Figlar and Nooteboom 2004) or divided into several smaller genera (Law 1984; Liu 2004; Xia et al. 2009). A good understanding of them would have important implications for revealing the origin of angiosperms and the systematics and evolution of the family Magnoliaceae (Xia et al. 2008; Wang et al. 2010). Understanding the genomic information of *M. yuyuanensis* would be necessary for conservation concerns, sustainable utilization, and taxonomy of this species. Chloroplast has been a valuable tool to be used for phylogenetic studies due to its gene conservation and the lack of recombination (Ravi et al. 2008; Lin et al. 2012). Here, the complete cp genome sequence of *M. yuyuanensis* was reported using next-generation sequencing. The annotated cpDNA has been deposited into GenBank with the accession number MN515039.

The fresh leaves were collected from three adult *M. yuyuanensis* plants from that were growing in Longzhong Botanical Garden (32°10′N, 112°10′E), Hubei, China. The specimen of *M. yuyuanensis* was stored in the Huazhong Agricultural University. The leaves were stored at −80 °C until used. Total DNA was extracted to construct a library for sequencing with Illumina Hiseq 2500 platform (Illumina, San Diego, CA, USA). Additionally, MITObim version 1.8 (https://github.com/chrishah/MITObim. . . . . . . . . .) was used to assemble the complete circular cp genome sequence (Hahn et al. 2013). The cp genome was annotated and manually adjusted with CpGAVAS (Liu et al. 2012). the circular plastid genome map was completed with the help of the online program OrganellarGenome DRAW (OGDRAW) (Lohse et al. 2013) and the annotated sequence was submitted to NCBI.

The complete cp genome sequence of *M. yuyuanensis* was 160,078 bp in length presenting a typical quadripartite structure and contains an inverted repeat region (IR, 26,467 b), a small single-copy (SSC) region, and a large single-copy (LSC) region (18,785 and 88,359 bp, respectively). The overall GC content was 39.27%. In total, 128 genes were annotated, including 81 (63.28%) protein-coding genes (PCGs), 38 (29.69%) tRNA genes, and 8 (6.25%) rRNA genes, one gene (0.78%) were inferred to be pseudogenes. Ten PCGs (*rps12*, *rpl2*, *trnV-UAC*, *trnL-UAA*, *atpF*, *ycf68*, *trnI-GAU*, *ndhA*, *trnA-UGC*, and *ndhB*) contained one intron, while *clpP* and *ycf3* each contained two introns.

For their phylogenetic placements within the family Magnoliaceae, phylogenetic analysis was conducted based on the maximum-likelihood (ML) analysis of the complete cp genomes of *M. yuyuanensis* with those of obtained from 30 other species of Magnoliaceae reported in Genbank of NCBI database using MEGA version7.0 (Kumar et al. 2016) (https://www.megasoftware.net. . . . . . . . . .). The phylogenetic analysis showed that *M. yuyuanensis* was closely related to *Manglietia glaucifolia*, forming a clade included in *Manglietia* (Figure 1). The genus *Manglietia* was phylogenetically closer to one another than to the other 23 taxa within the subfamily Magnolioideae. The cp genome of *M. yuyuanensis* will provide useful genomic resources for further study on genetic diversity and conservation of this species.

**Figure 1.**
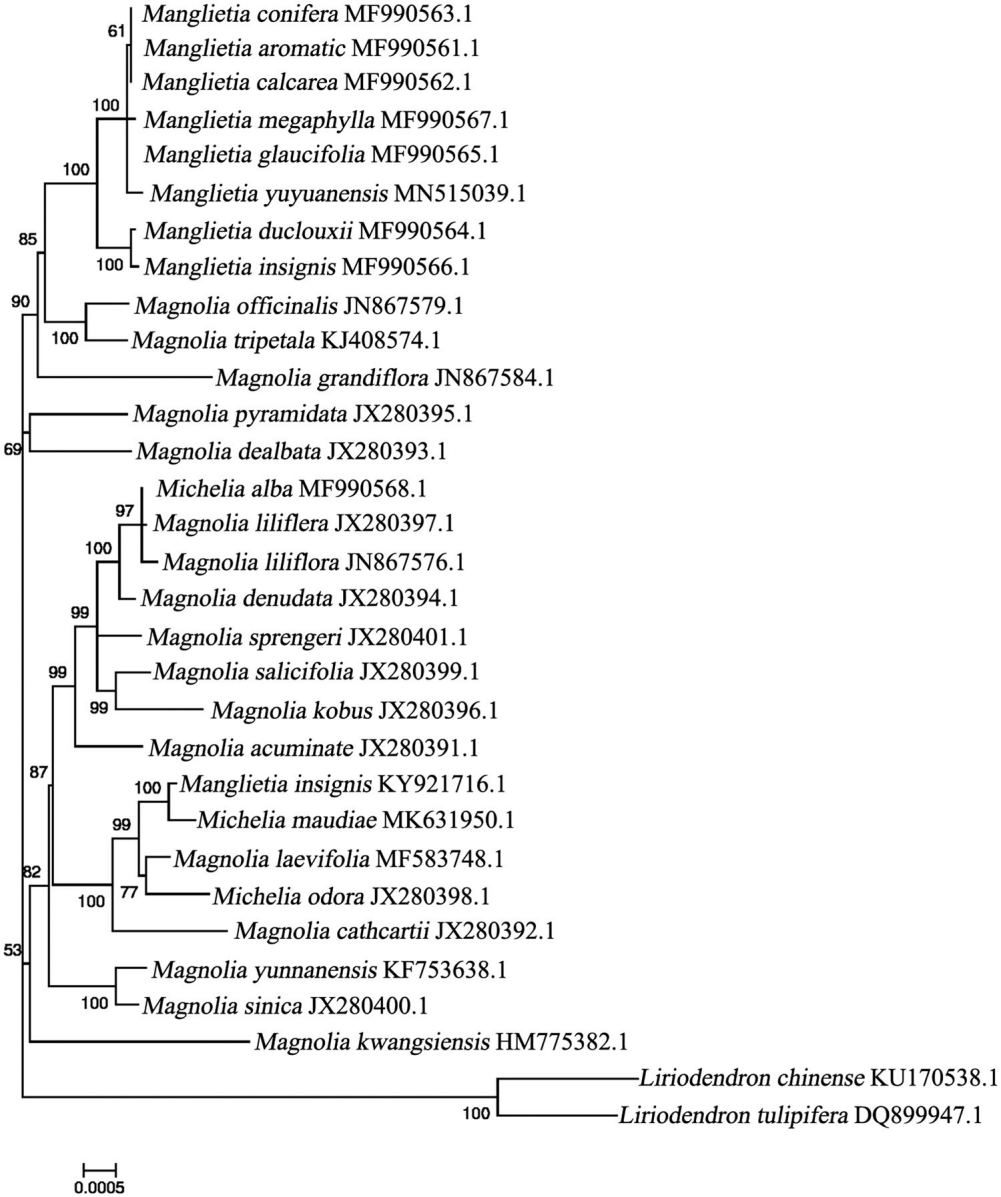
Maximum likelihood phylogenetic tree of 30 selected Magnoliaceae chloroplast genome sequences. Bootstraps (1000 replicates) are shown at the nodes.
